# Educating tomorrow’s doctors: A cross sectional survey of emotional intelligence and empathy in medical students of Lahore

**DOI:** 10.12669/pjms.293.3642

**Published:** 2013

**Authors:** Nazish Imran, Muhammad Awais Aftab, Imran Ijaz Haider, Anam Farhat

**Affiliations:** 1 Dr. Nazish Imran, MBBS; MRCPsych (London), Associate Professor, Child & Family Psychiatry Department, King Edward Medical University/Mayo Hospital, Lahore, Pakistan.; 2 Dr. Muhammad Awais Aftab, MBBS, House Officer, Child & Family Psychiatry Department, King Edward Medical University/Mayo Hospital, Lahore, Pakistan.; 3 Dr. Imran Ijaz Haider, MBBS; MRCPsych (London); DPM (England), Associate Professor, Department of Psychiatry& Behavioral Sciences, Fatima Memorial College of Medicine and Dentistry, Lahore, Pakistan. Child & Family Psychiatry Department, King Edward Medical University/Mayo Hospital, Lahore, Pakistan.; 4 Dr. Anam Farhat, MBBS, House Officer, Child & Family Psychiatry Department, King Edward Medical University/Mayo Hospital, Lahore, Pakistan.

**Keywords:** Emotional Intelligence, Empathy, Gender, Pakistan, Medical undergraduates

## Abstract

***Objective:*** Medical education in Pakistan traditionally emphasizes physician’s biomedical knowledge with less emphasis on interpersonal skills and ability to relate to the patients. This study explored the emotional intelligence & empathy of undergraduate medical students and investigated its relationship with various factors to act as baseline for future work in this area.

***Methodology:*** The Schutte Emotional Intelligence scale and Davis' Interpersonal Reactivity Index in addition to socio demographic questionnaire were administered to first year and final year medical undergraduates of two medical Institutions in Lahore, Pakistan. Data was analyzed by using SPSS 17 version.

***Results: ***The overall mean scores for medical students both on emotional intelligence and empathy is significantly lower than that found in previous literature, highlighting that medical students do not appear to fare better than average people in EQ. Women showed statistically significant higher scores on Appraisal of emotions , Regulation of emotions, Empathic concern Scale & Personal distress scale. Comparison of EI & IRI of students according to medical college year did not show any statistical significance.

***Conclusion: ***Current medical curriculum and training in Pakistan does not appear to increase EI abilities which are building blocks that may allow students and residents to develop competence. Medical educators in Pakistan should look for ways to incorporate emotional intelligence in medical curriculum which will ultimately contribute towards patient centered practice, patient satisfaction as well as effective communication skills.

## INTRODUCTION

Emotional Intelligence (EI) is "the set of abilities (verbal and non-verbal) that enable a person to generate, recognize, express, understand, and evaluate their own, and others, emotions in order to guide thinking and action that successfully cope with environmental demands and pressures".^1^ This concept was first described by Salovey and Mayer about a decade ago.^2^ High EI correlate positively with an individual’s academic success^3^^,^^4^ social skills,^5^ more cooperative interpersonal relationships and ability to cope with stressful situations,^6^ while low EI has been associated with deviant behavior, alcohol and drugs abuse and poor relationships.^7^^,^^8^

Emotional intelligence is a subject of growing interest in medicine and is considered among those non-cognitive factors which are considered to be desirable in future physicians to contribute towards patient centered practice, patient satisfaction as well as effective communication skills.^9^ In a study in which medical students wrote about their emotions in response to a hypothetical traumatic medical event, students whose writing reflected more emotional withdrawal and disengagement from the situation were later rated by standardized patients as having poorer specific and overall communication skills.^10^ EI is also suggested to be helpful in at least one admissions study to identify “applicants’ attributes most consistent with humanistic approach to medicine.^11^ Previous studies have also demonstrated some evidence for EI as a possible factor in mediating stress in healthcare students.^12^^,^^13^

The challenge in medical education is to understand and identify those psychological factors that help promote the development of effective skills, thereby allowing for the development of more effective curricula. Recently there have been demands to include training in EI in healthcare workers to improve leadership qualities, communication skills and prevent burnout, and stress. The EI abilities are building blocks that may allow students and residents to develop competence. The first step in applying an EI framework in medicine is successfully measuring EI in individuals.

Most of the studies related to emotional intelligence in medical education have been conducted in the developed world and this area is under researched in Pakistan. In order to formulate Emotional intelligence promoting curriculum for our country with its unique social, cultural and religious setup, the first step may be to determine the emotional intelligence of our medical students. The manuscript reports a pilot project looking at emotional intelligence of medical students in their first and final years of studies in two Medical Institutes in Lahore as a baseline for future work in this area. The years were chosen to determine if our current medical curriculum is effective in promoting Emotional intelligence skills as the student becomes more mature and competent in biomedical technical skills. 

## METHODOLOGY

The study was conducted in two medical institutions in Lahore for a period of approximately 6 months in 2011. Ethical Review Board of King Edward Medical University approved the study. All medical students in their first & final year of studies at both Institutions were invited to participate in the study. Written explanation of purpose of the study and emotional intelligence was given to the participants before they filled in the questionnaire and informed consent was sought. Questionnaire was anonymous to encourage participation. It was administered and collected immediately upon completion by the data collection team from the willing participants. The questionnaire was two paged, in English language & apart from demographic information contained Emotional Intelligence Scale & Davis' Interpersonal Reactivity Index (IRI).

The Emotional Intelligence Scale was developed by Schutte.^14^ It is a 33-item scale with a five-point Likert-type scale. The instrument has three categories: (a) the appraisal and expression of emotion assessed by 13 items; (b) the regulation of emotion assessed by 10 items; and (c) the utilization of emotion assessed by 10 items. Participants read each statement and decide whether they ‘strongly disagree’, ‘disagree’, are ‘undecided’, ‘agree’, or ‘strongly agree’ with the statement. Scores ranged from 33-165.

Davis’ IRI has also been successfully applied in medical student populations.^15^^-^^17^ It asks respondents to indicate their levels of (dis) agreement with 28 statements on a five-point Likert scale , related to four seven-item dimensions of empathy: (1) *Perspective Taking *is the tendency to embrace another’s point of view; (2) *Empathic Concern *is the regard or sympathy for another’s feelings; (3) *Personal Distress *is the response to another’s difficult interpersonal situations; and (4) *Fantasy *is the use of imagination to experience the feelings and actions of characters in creative works. Only the first three dimensions were used in this study. The subscale scores range from 0-28 and summation of subscale scores is not recommended as they are not positively correlated.^18^

Data was analyzed by using SPSS 17 version. Descriptive statistics were computed for the whole data. EI scores and empathy scale scores were compared between the two medical year’s students as well as between genders by using the appropriate test. Statistical significance was fixed at level of P<0.05.

## RESULTS

Total sample size was 443/615 students (response rate 72.0) & majority being female. The mean scores of respondents of the two Institutions on EI and IRI subscales are given in Table-I.

**Table-I:** Demographic details and Mean EI & IRI scores of the sample. (Total N=443

	*Institution # 1(N=327)*	*Institution #2(N=116)*
*Age (Mean; sd)*	19.8(2.10)	20.6(2.2)
Gender (N, %)		
Female Male	215(65.7%) 103(31.5%)	84(72.5%) 29(25.0%)
*Year of Medical College * *(N, %)*		
First Year Final Year	211(64.5%) 115(35.2%)	71(61.2%) 42(36.2%)
*Emotional Intelligence Score. * *Mean(95%CI)*		
Total score Appraisal of emotions Regulation of emotions Utilization of emotions	122.4(120.5-124.4) 46.8(45.9-47.8) 37.0(36.3-37.7)) 38.5(37.8-39.3)	123.3(120.6-126.0) 45.9(44.6-47.1) 37.5(36.6-38.5) 39.9(38.9-41.0)
*Interpersonal Reactivity Index (IRI).Mean(95%CI)*		
Empathic concern Scale Perspective Taking Scale Personal distress scale	20.0(19.7-20.8) 15.6(15.0-16.3) 15.0(14.3-15.7)	19.2(18.4-20.0) 16.64(15.7-17.5) 14.56(13.5-15.6)

Comparison of EI & IRI of students according to gender is presented in Table-II. Women showed statistically significant higher scores on Appraisal of emotions, Regulation of emotions, Empathic concern Scale&Personal distress scale.

**Table-II:** Comparison of EI & IRI of students according to gender

	*Female* *N=299* *Mean(95%CI)*	*Male* *N=132* *Mean(95%CI)*	*P- Value*
*Emotional Intelligence(EI) Total score* Appraisal of emotions Regulation of emotions Utilization of emotions	123.4(121.5-125.2) 46.8(45.9-47.7) 37.6(36.9-38.2) 39.0(38.3-39.7)	121.4(118.4-124.3) 45.8(44.4-47.2) 36.3(35.3-37.4) 39.1(37.9-40.4)	0.48 0.04* 0.05* 0.46
*Interpersonal Reactivity Index (IRI).*			
Empathic concern Scale Perspective Taking Scale Personal distress scale	20.2(19.7-20.7) 16.2(15.6-16.8) 15.4(14.7-16.1)	19.2(18.3-20.0) 15.3(14.4-16.3) 13.5(12.4-14.5)	0.007* 0.67 0.03*

*indicates significant P value<.05

Comparison of EI & IRI of students according to medical college year did not show any statistical significance. (Table-III)

**Table-III:** Comparison of EI & IRI of students according to medical college year

	*First Year* *N=282* *Mean(95%CI)*	*Final Year* *N=157* *Mean(95%CI)*	*P- Value*
*Emotional Intelligence(EI) Total score* Appraisal of emotions Regulation of emotions Utilization of emotions	122.7(120.7-124.7) 46.5(45.5-47.4) 37.0(36.3-37.7) 39.1(38.4-39.9)	122.7(122.0-125.3) 46.5(45.2-47.7) 37.5(36.6-38.3) 38.7(37.7-39.8)	0.26 0.47 0.27 0.56
*Interpersonal Reactivity Index (IRI).*			
Empathic concern Scale Perspective Taking Scale Personal distress scale	19.7 (19.1-20.2) 15.7(15.0-16.3) 14.8(14.1-15.5)	20.3 (19.6-21.0) 16.5(15.6-17.3) 14.9 (13.9-15.8)	0.90 0.26 0.49

*indicates significant P value<.05

## DISCUSSION

Emotional Intelligence is an interesting and appealing concept which appears to be relevant to patient centered care in the field of medicine. Doctors should have empathy, should be active listeners, should be able to understand different point of views and at the same time they need to be able to understand their own reactions as well as handle stress in an appropriate way, and almost all of these relates to the concept of Emotional intelligence.

We found total EI, almost similar in both groups in first year and final year suggesting a similar level of gross emotional functioning in the medical students in our sample. This raises two important points. First is that the overall mean scores for medical students both on emotional intelligence and empathy is significantly lower than that found in literature highlighting that medical students do not appear to fare better than average in EQ which is considered the case in IQ. Stratton et al^19^ in a study of medical students found mean scores of 26.9, 26.6 and 15.0 on subscales of perspective taking, empathic concern and personal distress versus much lower mean of approximately 20.0, 15.6 and 15.0 found in our medical students. The EI scale original validation paper cited mean scores for therapists (134.9), prisoners (120.8) and clients in substance abuse treatment (122.2), which again compared with our students mean scores of 122.4 also do not appear promising, to say the least.^14^ Whether the scores constitute meaningful clinically relevant changes remains unclear but evidence is for students having higher EQ doing well in clinical skills.^19^ Wagner and her colleagues also documented a modest correlation between physicians’ EI and patient satisfaction.^20^

Second perhaps more surprising aspect is that EI of students do not appear to increase as the experience in medical college increases. In a study to examine changes in emotional scores across undergraduate medical curriculum, empathic concern, attention to feelings and mood repair were noticed to be lower than at the baseline while personal distress was found to be higher.^21^ No significant gender differences were observed. At the moment most students in Pakistani medical colleges are admitted on basis of their academic competence and performance rather than their desirable EQ contributes. Also unfortunately current medical training focuses more on hard skills training and technical aspects of medicine with less emphasis on soft skills like interpersonal skills and ability to relate to patients. Medical curriculum needs to be modified to put more emphasis on interpersonal skills. It will also help to select applicants in medical college with high EQ.^22^

Comparison of EI & empathy scores by gender in our study showed statistically significant differences in few subscales with trend of women scoring slightly better. (Table-II) Previous literature search found some studies with females scoring higher on EI.^23^ On the contrary another study demonstrated higher self-estimated EI in males than females^24^ and few reported no gender differences.^12^^,^^21^^,^^25^ One possible reason for results may be the difference in culture as psychological properties have been noticed to be different in males and females belonging to different cultures.^26^ Women are innately more receptive than men to emotional signals, which can explain better empathy and EI.

Given the positive association of EI with desirable competence in medicine, it is important to take steps to enhance it, one of which is by practicing skills which make up EI.^27^ Systematic review of emotional intelligence training identified 5 randomized controlled trials all showing positive outcomes with improvement in empathy, communication skills, better patient understanding and supportive behavior.^28^ All courses varied in duration, frequency of sessions & educational outcomes etc. but effectiveness of all training methods was clearly demonstrated. Some medical colleges like MC of Georgia by use of small group teaching as part of Essentials of clinical medicine (ECM) course focuses on increasing their medical students understanding of their own emotional skills with view to help them improve the patient care ultimately.^29^ Medical educators of today know that our students learn many things via “informal curriculum” in ward rounds, lectures and thus presence of good role models also help in the task of improving EI. Furthermore we should explore ways to incorporate mindfulness in our medical curriculum to help produce emotionally intelligent physicians.^30^

Research on EI is still in its infancy in Pakistan. Although as far as we are aware this is the first study of its kind in Pakistan and important but still the results of the study should be seen as largely exploratory & in the context of its limitations. The study is preliminary, sample size is small. We used cross sectional method due to our resource limitation; longitudinal design may have been more helpful. Furthermore repeated assessment of first year students again when they are in final year and again as practicing physicians would also help to answer if students with higher EI show better performance in clinical skills. Also, we used self-reported measure of EI which given the nonverbal nature of the construct of EI may not validly reflect the qualitative nature of the EI. It is also susceptible to faking good. Also we should consider the cultural bias in use of these scales despite the fact that they have been used intensively in various studies conducted worldwide. Although self-reported measures have been shown in research to correlate more strongly with personal & performance measures but still it would be helpful to compare EI using different means including trait and performance measures to predict objective performance ability.

## CONCLUSION

In conclusion, healthcare needs, expectations and demands of population all over the world including Pakistan are changing and so should the medical education. Unfortunately it looks like that our current medical curriculum and training does not appear to increase EI. We believe that the first step is for students to examine and understand their own EI, which will assist them in ability to identify and accept patients and their caregiver’s point of views. Medical educators in Pakistan should look for ways to incorporate building blocks of emotional intelligence in our curriculum. EI instrument can be used as an adjunct to admission process to measure desirable personal attributes in future physicians which can be further nurtured during medical training. As we prepare doctors to meet society’s expectations in twenty first century we must remember that there is no substitute for a doctor’s professionalism and competence,& EI being an integral part of it.

## Author’s contributions

*NI* conceived the study, wrote the literature review, participated in its design and coordination, analyzed the data and helped to draft the manuscript. *MAA* participated in design of the study, collected the data and helped in analysis. *IIH* helped in design, data collection, drafting of manuscript & critical revision. *AF* helped in data collection & critical revision. All authors read and approved the final manuscript.

## Notes

***List of Abbreviations: ***EI (Emotional Intelligence; IRI (Interpersonal Reactivity Index); ECM (Essentials of clinical medicine).
